# Comparative Genomic Analysis of Prophages in Human Vaginal Isolates of *Streptococcus agalactiae*

**DOI:** 10.3390/pathogens13080610

**Published:** 2024-07-23

**Authors:** Caitlin S. Wiafe-Kwakye, Andrew Fournier, Hannah Maurais, Katie J. Southworth, Sally D. Molloy, Melody N. Neely

**Affiliations:** 1 Department of Molecular and Biomedical Sciences, University of Maine, Orono, ME 04469, USAsally.dixon@maine.edu (S.D.M.); 2 The Honors College, University of Maine, Orono, ME 04469, USA

**Keywords:** prophage, group B Streptococcus, comparative genomics

## Abstract

Prophages, viral genomes integrated into bacterial genomes, are known to enhance bacterial colonization, adaptation, and ecological fitness, providing a better chance for pathogenic bacteria to disseminate and cause infection. *Streptococcus agalactiae* (Group B Streptococcus or GBS) is a common bacterium found colonizing the genitourinary tract of humans. However, GBS-colonized pregnant women are at risk of passing the organism to the neonate, where it can cause severe infections. GBS typically encode one or more prophages in their genomes, yet their role in pathogen fitness and virulence has not yet been described. Sequencing and bioinformatic analysis of the genomic content of GBS human isolates identified 42 complete prophages present in their genomes. Comparative genomic analyses of the prophage sequences revealed that the prophages could be classified into five distinct clusters based on their genomic content, indicating significant diversity in their genetic makeup. Prophage diversity was also identified across GBS capsule serotypes, sequence types (STs), and clonal clusters (CCs). Comprehensive genomic annotation revealed that all GBS strains encode paratox, a protein that prevents the uptake of DNA in Streptococcus, either on the chromosome, on the prophage, or both, and each prophage genome has at least one toxin-antitoxin system.

## 1. Introduction

*Streptococcus agalactiae* (group B Streptococci or GBS) is a commensal pathogen found on the mucus membranes of the intestinal and vaginal tracts in humans [1,2]. Rectovaginal colonization of pregnant women is a major risk factor for neonatal GBS disease [3]. Approximately 50% of babies born through vaginal delivery from women colonized with GBS develop life-threatening infections such as meningitis and sepsis [4]. Recommended infection management includes antibiotic treatment of newborns after delivery and intrapartum antibiotic prophylaxis (IAP) for mothers immediately prior to and during delivery. Although commonly used, IAP does not address the risk of infection in utero nor when the infection presents in babies over 7 days old [5]. Additionally, antibiotic treatment of newborns has long-term negative effects on neonatal microbiota, affecting not only metabolism and nutrition but also postnatal development of the immune system [6,7,8,9]. These long-term effects, in addition to the rise in antibiotic and multi-drug resistance in GBS [10], highlight an increasing need for alternative therapeutic approaches to reduce vaginal colonization and treat neonatal infections. Recent studies have uncovered multiple factors that contribute to GBS colonization and virulence [5,11,12,13,14,15,16], providing new insights into the development of effective treatments and preventive measures.

The successful colonization of GBS on the vaginal epithelium relies on factors like the capsular polysaccharide (CPS), of which 10 different serotypes have been described (Ia, Ib and II-IX). Six of these capsular serotypes (Ia, Ib, II, III, IV, and V) are most commonly associated with disease in humans [17]. GBS is further classified into clonal complexes (CCs) based on their sequence type (ST), determined by sharing at least five of the seven MLST (multi-locus sequence typing) loci [17,18]. Some CCs are linked to invasive disease while others primarily colonize pregnant women [19,20]. In addition to chromosomal virulence factors, the GBS genome contains diverse adaptable genetic elements like prophages, plasmids, insertion sequences, and transposons, which facilitate mutations and lateral gene transfer, ultimately enhancing GBS pathogenicity and ability to survive in different environments [21,22].

Prophages, viral genomes integrated into the bacterial chromosome, can enhance bacterial colonization, environmental adaptation, and ecological fitness, increasing the opportunity for pathogenic bacteria to disseminate and cause infection [20,23]. Phage infection dynamics can drive horizontal gene transfer in bacteria, allowing them to adapt to challenging environments [23]. Prophages can also change the structure of the bacterial genome by functioning as sites for genomic rearrangements or acting as vehicles for the horizontal transfer of bacterial genes [24,25]. Most genomes of GBS contain one or more prophages [26,27,28,29], yet their role in bacterial fitness and virulence has not yet been described.

Here we report the sequences and diversity of GBS prophages found in 49 GBS human isolates, including their distribution within serotypes and clonal complexes. GBS prophage genomes were analyzed for genetic content and organization and were sorted into clusters based on genomic similarity [30,31]. The integration sites and genetic composition of prophage genomes were analyzed for their potential to impact bacterial gene expression and host fitness. Prophage genes that potentially benefit the bacterial host were identified. One such potential beneficial phage protein is paratox, previously identified in prophage genomes of *Streptococcus pyogenes* [32], where it is proposed to prevent the uptake of DNA. Paratox is strictly a prophage-encoded protein in *S. pyogenes*; however, in GBS we find paratox encoded on the bacterial chromosome of all human isolates, possibly as a remnant of a mobile genetic element. A second copy of paratox is maintained in GBS prophage genomes, suggesting the importance of the conservation of this protein to GBS.

## 2. Materials and Methods

### 2.1. Bacterial Strains and DNA Isolation

GBS human isolates collected from the vaginal tracts of pregnant women at Detroit Medical Center [18] were used in this study. All strains were de-identified from patients. Genomic DNA was extracted from GBS samples for whole genome sequencing. Briefly, 10 mL overnight cultures grown at 37 °C were pelleted and resuspended in 1 mL of TE (10 mM Tris, 1 mM EDTA, pH 8.0) with 10 µL of 25 mg ml^−1^ of lysozyme and 5000 U mL^−1^ mutanolysin, and incubated at 37 °C for 1 h. Cell pellets were then subjected to a freeze–thaw process (–80 °C for 5 min, 37 °C for 5 min) and resuspended in 800 Nuclei Lysis solution (Wizard Genomic DNA purification kit, Promega, Madison, WI, USA). Cells were incubated for 5 min at 80 °C to lyse the cells and then cooled at room temperature. After treatment with RNase solution (3 µL of 10 mg mL^−1^) for 15 min at 37 °C, the sample was cooled to room temperature and protein precipitation solution (Wizard Genomic DNA purification kit, Promega, Madison, WI, USA) was added to the RNase-treated cell lysate. The DNA was precipitated, rehydrated with 100 µL of DNA rehydration solution (Wizard Genomic DNA purification kit, Promega, Madison, WI, USA), and incubated at 65 °C for 1 h. The isolated DNA was sent to the Hubbard Center for Genome Studies (HCG) (Durham, NH, USA) for whole genome sequencing. Previously published genome sequences of seven GBS clinical isolates were included, namely 2603 V/R (NC_004116.1), 515 (NZ_CP051004), A909 (NC_007432), CJBIII (NZ_CP063198), CNCTC 10/84 (NZ_CP006910), COHI (NZ_HG939456), and NEM316 (NC_004368.1).

### 2.2. Genome Sequencing, Assembly, and Prophage Isolation

Whole-genome sequencing libraries were prepared following the Kapa BioSystems HyperPlus Kit (KR1145-v3.16). Sequencing was completed at the Hubbard Center for Genome Studies (Durham, NH) on an Illumina NovaSeq 6000 and produced 250 bp paired-end sequencing reads. Sequencing data were demultiplexed using the Illumina bcl2fastq Conversion Software v1.8.4. Sequenced DMC isolates were assembled and annotated using a sequential list of programs (GitHub tutorial https://github.com/Joseph7e/MDIBL-T3-WGS-Tutorial; accessed on 4 September 2020) organized by Kelley Thomas at the HCG. The sequenced raw reads were quantified using basic BASH commands. The quality of the raw reads was examined using FastQC v0.11.5 (accessed on 4 September 2020) [33] and exported as HTML figures. Low-quality base reads and adaptors were removed using Trimmomatic v0.36 [34] and exported as paired forward, paired reverse, and unpaired forward and reverse FASTQ files. Genome assembly was performed using SPAdes v3.11.0 [35] to assemble the trimmed read files in a de novo fashion. The resulting contiguous sequences were quantified and organized by length. The program QUAST [36] was used to assess overall genome structure and ensured contiguity of the assembled reads. Genomic content was assessed with the program BUSCO [37], which examined the contiguous sequences for common single-copy bacterial orthologs. Each sequence was annotated using PROKKA v.14.6 [38], which individually examined DNA coding sequences, rRNA, tRNA, and ncRNA. Ribosomal RNA sequences were compared against the BLAST nucleotide database [39] to confirm samples as *Streptococcus agalactiae*. Read mapping was performed to calculate the coverage of each contig using BWA-MEM v0.7.17 [40] and SAMtools v0.1.20 [41]. The program Blob_tools v1.1.1 [42] BLASTed each contig against a complete nucleotide database to create a taxonomy table. The taxonomy table was filtered according to length (>500 bp), GC content (between 30% and 50% GC), coverage (>4), and, in some cases, species identification (*S. agalactiae*). Heavily contaminated samples were rejected. Filtered contigs were parsed against PHASTER [43] for putative prophage regions. All prophage genomes were manually examined in Geneious Prime 2021.2 (https://www.geneious.com; accessed on 27 July 2020) for defined genome ends.

### 2.3. GBS Prophage Database Creation and Genome Clustering

To create the GBS prophage database, GenBank flat files of all prophage genomes were submitted to Dr. Steven Cresawn of James Madison University as input files to be uploaded to the Phamerator website (https://phamerator.org; uploaded on 23 February 2022). Multiple techniques were employed, including EMBOSS’s polydot function [34] for dot plot analysis, and gene content analysis to identify prophage clusters, as previously described [30,31]. Direct comparisons of genomes within and across clusters were performed by visualizing genome maps in Phamerator *Streptococcus* database version 1 [44].

### 2.4. Genomic Analysis of Prophages

Prophages were auto-annotated using GLIMMER v3.02 and GeneMark v2.5 within DNA Master v5.23.6 (http://cobamide2.bio.pitt.edu; accessed on 16 January 2018) and PECAAN (http://pecaan.kbrinsgd.org; first accessed on 25 January 2018) [45,46]. Translational starts were predicted manually based on GeneMark.hmm and conservation across homologs in BLAST, and putative gene functions were predicted using BLAST, TMHMM 2.0, and HHpred [39,47,48]. Schematic diagrams of bacterial genes flanking the prophage region and genes surrounding the bacterial paratox were produced in Geneious Prime 2021.2 (https://www.geneious.com; accessed on 27 July 2020). Clustal alignment was performed using clustalw [49]. Graphs were generated with RStudio [50] and Python 3.9.5 using the packages Matplotlib 3.5.1 [51], pandas 1.4.2 [52], NumPy 1.22.4 [53], and seaborn 0.11.2 [54]. All figures were edited with Inkscape (https://inkscape.org; accessed 2 March 2021).

## 3. Results

### 3.1. Identification of Prophages in GBS Clinical Isolates

The distribution of prophages in GBS human isolates was determined by sequencing the genomes of 42 strains collected from the vaginal tracts of pregnant women at the Detroit Medical Center (DMC) in Detroit, Michigan. These 42 GBS strains were previously examined for virulence potential [18]. High-coverage draft genome sequences of the 42 human vaginal isolates were obtained with an average genome size of 2.03 Mbp (Supplementary Table S1). Additionally, prophages were identified in the genome sequences of seven previously published clinical isolates; 2603 V/R (NC_004116.1), 515 (NZ_CP051004), A909 (NC_007432), CJBIII (NZ_CP063198), CNCTC 10/84 (NZ_CP006910), COHI (NZ_HG939456), NEM316 (NC_004368.1) and used as reference strains (Table 1). Of the seven previously published clinical isolates, six were obtained from the blood of neonates, with the exception of 515 (NZ_CP051004), which was isolated from cerebrospinal fluid (CSF). Analysis of the 42 strains in this study confirmed previous reports that serotypes III and V account for half of the strains [18]. In addition, seventeen sequence types and ten major clonal complexes were identified, as presented in Supplementary Table S2.

A total of 75 prophage regions were identified from the human vaginal isolates, of which 36 full-length prophages were extracted. Out of the full-length prophages, 80% (28/36) were extracted from a single contig and 20% (7/36) from two contigs in the sequenced vaginal isolates (Supplementary Table S1). Prophages extracted from two contigs were manually inspected to ensure that the genome was complete and confirmed with PCR In addition, seven prophages were identified in the previously published genomes, out of which four had been previously identified (Javan 5 and Javan 6 in 2603V/R, and Javan 7 and Javan 8 in A909) [55]. Altogether, a total of 43 prophages were extracted from the 49 GBS human isolates analyzed in this study. The prophage genomes identified in the DMC vaginal isolates were designated with prophiDMCx-#, where x is the name of the bacterial strain and # is the number assigned to distinguish multiple prophages that exist in a single bacterial strain. Out of the GBS genomes examined, most (69.4%, 34/49) had only one prophage, while 12.2% (6/49) had two prophages, and 18.3% (9/49) had no prophages in their bacterial genome (Supplementary Table S1). Among the 43 prophages identified, 4 were found to be identical, resulting in a total of 39 unique prophage genomes.

### 3.2. Prophages Are Prevalent across GBS Serotypes and Clonal Complexes

**Figure 1:**
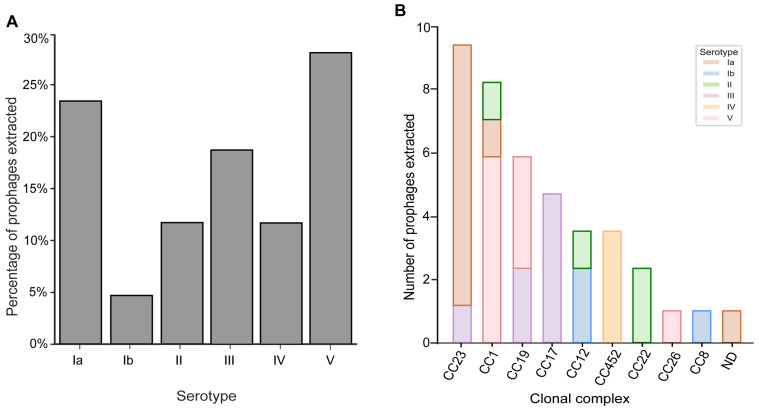
Prophage distribution. (**A**) Prophage distribution across GBS serotypes identifies in our sample collection. (**B**) Serotype relationship and prophage distribution within individual clonal complexes (CC).

GBS strains can produce one of eleven different capsular serotypes (Ia, Ib-X). Specific GBS capsular serotypes have been associated with clinical disease and virulence; therefore, the distribution of prophages across the different serotypes of the GBS vaginal isolates was examined. Serotype V and Ia accounted for 30.2% (13/43) and 23.2% (10/43) of the extracted prophages, respectively (Figure 1A). These serotypes are usually associated with adult infections [56] and account for more than half of the extracted prophages (Figure 1A). Prophages extracted from strains belonging to serotype III, a serotype commonly implicated in neonatal infections [57], constituted 18.6% (8/43) of all prophages, with the remaining belonging to serotypes II (11.6%, 5/43), IV (11.6%, 5/43) and Ib (4.6%, 2/43) (Figure 1A).

GBS strains are segregated into sequence types (STs) that share the same sequence of seven housekeeping genes. Sequence types are then further grouped into clonal complexes (CCs) based on the sharing of five or more alleles of the same seven loci. A particular CC is designated after its ancestor ST or the predominant ST within the clone [58]. To determine if there is a relationship between prophage carriage and specific clonal complexes, we evaluated the number of prophages extracted within individual clonal complexes. Over half of the prophages were extracted from CC1 (23.2%, 10/43), CC23 (20.9%, 9/43), and CC19 (13.9%, 6/43) (Figure 1B), clonal complexes common for invasive GBS disease and consistently reported in asymptomatic pregnant women [20].

**Figure 2:**
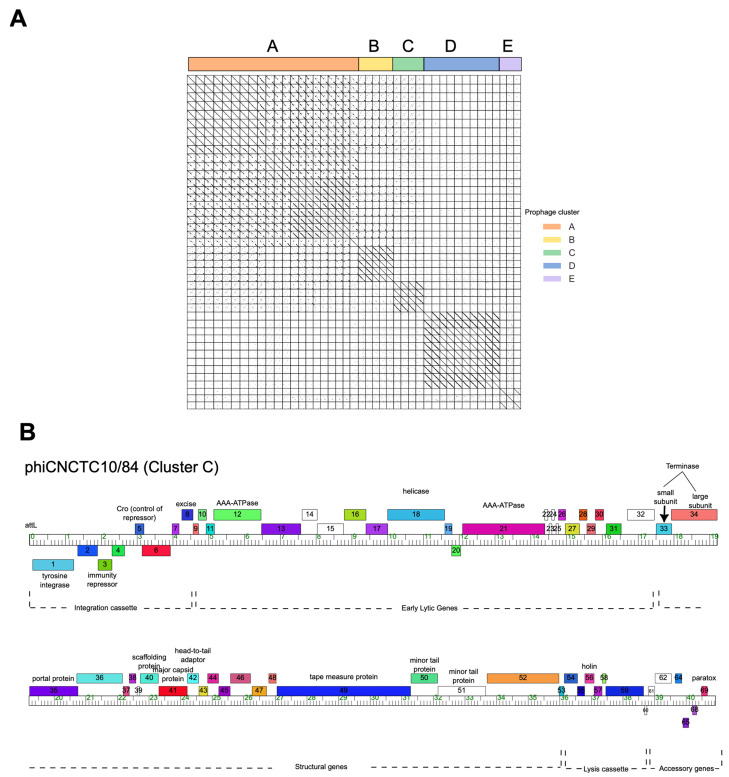
Prophage diversity. (**A**) Nucleotide sequence comparison of 43 GBS prophages from whole genome sequences concatenated into a single file and compared with itself using polydot (EMBOSS; word size, 15). Dotplot analysis identified five distinct prophage clusters. (**B**) Genome map of Callidus showing genome organization with genes represented as boxes above and below the ruler illustrating genes transcribed in the forward and reverse directions, respectively. The genome coordinates are represented by the ruler in units of kilobase pairs. Genes are colored according to assigned ‘phamilies’ with putative gene functions indicated above the genes.

To better understand the diversity of the prophage genomes, gene content and genome organization were compared across the 43 prophage genomes. Based on more than 50% nucleotide sequence similarity and shared gene content of over 35%, as done previously in mycobacteria prophages [31], the GBS prophages can be sorted into five distinct clusters. These five clusters were randomly assigned the letters A–E (Figure 2A; Supplementary Table S2). In cluster A, there are a total of 21 prophages, while cluster B contains 5, cluster C has 4, cluster D has 10, and cluster E has 3 prophages. Prophage genome size ranges from 34,100 to 48,336 bp in length, with prophages of clusters E and A having the shortest [35,512 bp] and longest [45,615 bp] average genome length, respectively. Cluster A prophages have an average genome length of 42,433 bp but contain the widest range in genome sizes (Supplementary Figure S1A). Clusters A and C had a similar %GC content to their bacterial host genome (35.6%), whereas clusters B, D, and E had a higher %GC content ranging between 39 and 43.9% (Supplementary Figure S1B). No clear relationship was found between prophage clusters and GBS serotypes or clonal complexes, except for cluster E prophages, which were only found in serotype V strains (Supplementary Figure S1C,D).

A genome map of prophages allowed for visualization of the genetic diversity within and across prophage clusters (Supplementary Figure S2A–E). Prophages within each cluster have the same organization, with the left arm encoding the immunity cassette and the early lytic genes and the right arm encoding structural and lysis genes (Figure 2B). Many prophages also encode accessory genes, located on the far-right arm downstream of the lysis cassette, that are most likely expressed during lysogeny. The prophage genomes of clusters B, C, D, and E are highly conserved within the cluster, whereas prophage genomes of cluster A are more diverse, especially in regions encoding early lytic genes (Supplementary Figure S2A–E).

### 3.3. Prophages Integrate within Specific Regions of Their Streptococcal Host Genome

**Figure 3:**
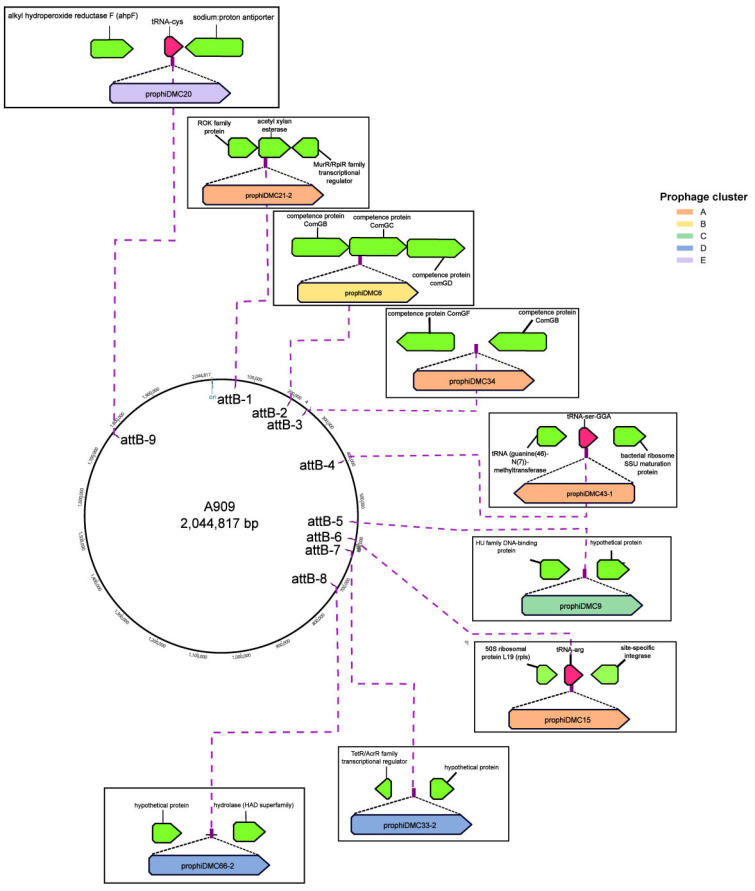
Prophage insertion locations on GBS. Nine different prophage insertion sites were identified based on the attachment site sequence and is displayed on GBS A909 as a reference genome. Attachment sites are numbered based on their location from the site of origin. Each box shows site of insertion and flanking chromosomal genes. Genes are colored green and tRNA are colored pink. Representative prophages that use the insertion site are shown and shaded according to cluster.

Integration of bacteriophages into the bacterial genome occurs at specific attachment sites (att sites), which are identical, short DNA sequences found in both the bacterial and phage genomes. These sites serve as points of recombination during integration [59]. The bacterial attachment sites (attB) for the GBS prophages were identified and mapped to the GBS reference genome, A909 (Figure 3). The prophages found in GBS are inserted at nine different locations across the genome, indicating a broad distribution (Figure 3, Table 1). Prophages can encode one of two types of integrases—tyrosine or serine. Twenty-five out of forty-three prophages identified encoded tyrosine integrases (Int-Y), and the remaining eighteen encoding serine integrases (Int-S). Prophages from clusters A, C, and E encode tyrosine integrases. Clusters A and E integrate into a tRNA gene with one exception (Javan 7), while cluster C integrates into intergenic regions. Prophages from clusters B and D, and three members of cluster A, encode serine integrases and integrate into a protein coding gene or into intergenic regions (Figure 3, Table 1).

Most cluster A prophages use an attB site (attB-6) located within a bacterial host arginine tRNA, except for prophiDMC43-1 which is located within a bacterial host tRNA-Ser (GGA) (attB-4). Cluster E prophages use a different attB site (attB-9) located within a host cysteine tRNA. The common core sequences shared by attB and attP are typically 12–18 bp for cluster A and 16 bp for cluster E, with phage-derived sequences reconstructing the 3’ end of the bacterial host tRNA gene. Int-Y prophages integrating at attB-5, a non-tRNA attB site, have a longer core sequence of 28 bp, and these are used by cluster C prophages and a single cluster A prophage (Javan 7). This attB site is located between a hypothetical protein and an HU family DNA-binding protein.

Five attB sites (attB-1, attB-2, attB-3, attB-7, and attB-8) are used by Int-S systems with common core sequences between 10 and 15 bp. Two of the five attB sites are found within open reading frames which they disrupt. Cluster B prophages integrate at attB-2 within the *com* gene locus, as described similarly for the ϕ10403S prophage of *Listeria monocytogenes* [60], and a single cluster A prophage (prophiDMC21-2) integrates at attB-1 within a gene that encodes acetyl xylan esterase, one of the accessory enzymes for xylan degradation. The remaining three Int-S attB sites are located in intergenic regions, specifically between ComGF and ComGB (attB-3), a transcriptional regulator AcrR family protein and a hypothetical protein (attB-7), and a hypothetical protein and HAD family hydrolase (attB-8). Cluster D prophages use attB-7 and attB-8, while attB-3 is used by two cluster A prophages.

### 3.4. GBS Prophages Encode Multiple Toxin-Antitoxin Systems

**Figure 4:**
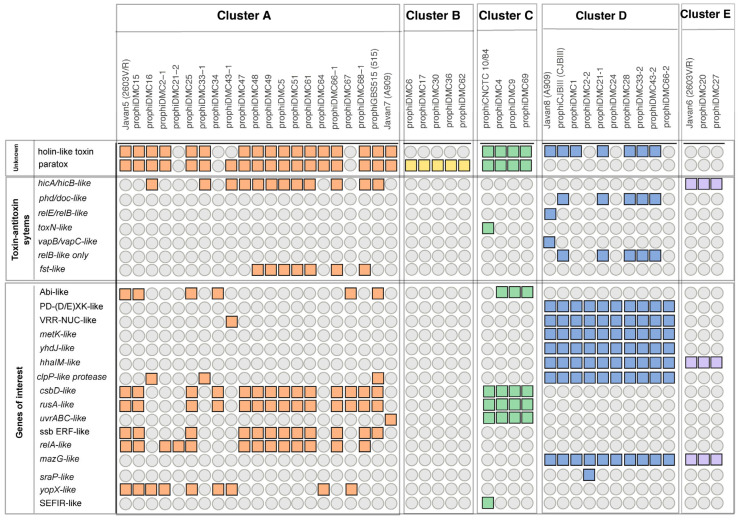
Identified genes that may contribute to bacterial fitness. Genes of interest are widely dispersed among clusters. Co-occurrence of the paratox and holtox proteins is diverse and not cluster dependent. Solid colored boxes represent the presence of genes of interest by prophage cluster; orange-cluster A, yellow-cluster B, green-cluster C, blue-cluster D, purple-cluster E. The absence of a gene is indicated by a grey circle.

Prophages encode genes that contribute to bacterial survival in several pathogens such as Vibrio cholerae, enterohemorrhagic Escherichia coli (EHEC), and Streptococcus pyogenes [24]. However, the role of prophages in GBS is not well understood. GBS prophage genomes are enriched with genes that potentially contribute to bacterial fitness, including toxin–antitoxin (TA) systems. TA systems are genetic modules composed of a toxin and its cognate antitoxin, where the toxin can either inhibit growth or kill the bacterial cell, and the antitoxin can neutralize these effects. These systems play roles in stress response, plasmid maintenance, and the regulation of bacterial growth and death [61]. These TA systems were present in prophages of all clusters except cluster B (Figure 4). TA systems identified were unique to specific clusters. For example, about 30% of cluster A prophages have the fst-like TA system, believed to have a role in bacterial adaptation to adverse environmental conditions, promoting survival in harsh or fluctuating environments [62], while cluster A and cluster E phage genomes have the hicAB systems that target cellular RNAs [63]. Half of the cluster D prophages have phd/doc-like genes, a type II TA system thought to have a role in maintaining the stability of prophages within bacterial genomes [64]. The cluster D prophage, Javan 8, has both relE/relB-like and vapB/vapC-like gene cassettes, which are type II TA systems that inhibit translation [65]. Several cluster D prophages also encode RelB-like antitoxin, without its cognate toxin RelE, and no relE homolog was found in these prophages. Only one cluster C prophage, prophiCNCTC10/84, has a homolog of the toxN gene, the toxin of a type III TA system believed to be involved in phage defense [61] with its cognate RNA pseudoknot upstream of the toxN gene (Figure 4).

### 3.5. Prx Is Encoded on the Bacterial Chromosome and Often in GBS Prophages

**Figure 5:**
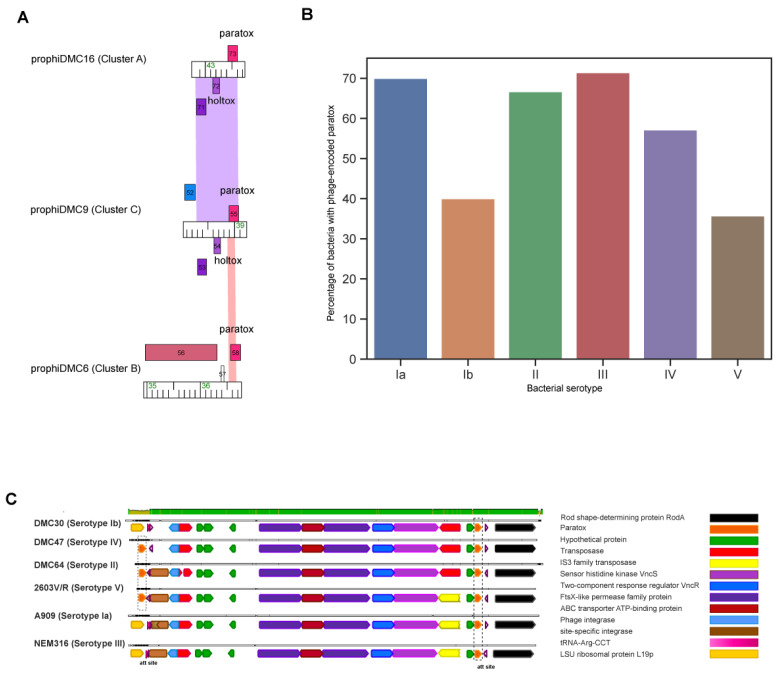
Paratox is encoded from both the prophage genome and the bacterial chromosome. (**A**). Phamerator map alignment of prx gene from selected prophages within each cluster (**A**–**C**). (**B**,**C**). Alignment of genome region of prx genes found on the host chromosome. The color panel above the selected sequences indicates high-to-low sequence conservation. Grey blocks indicate homologous sequences. Genes are marked by arrows, for which the putative functions are indicated by the colored key. Dotted box indicates prx gene.

A notable feature identified in most GBS prophages is the presence of the *prx* gene, encoding the protein paratox. Paratox is a conserved protein in multiple streptococcal species including *Streptococcus pyogenes*, *Streptococcus dysgalactiae*, *Streptococcus equi*, and GBS [32]. In *S. pyogenes*, *prx* is strictly encoded on the distal right arm of prophage genomes. Mashburn-Warren et al. demonstrated that paratox prevents cellular uptake of DNA by inhibiting the ComR protein, a component of the quorum sensing ComRS system that triggers competence in Streptococcus [32]. When analyzing the GBS prophage genomes for a homolog of *prx*, more than 60% (27/43) of the GBS prophages across clusters A, B, and C contain a *prx* homolog (~98.3% identity) located at the right terminal end of the prophage, adjacent to the phage attachment site (Figure 4 and Figure 5A).

The paratox protein is highly conserved across prophage genomes from clusters A, B, and C, although there is a 3-amino acid difference in the C terminus among cluster B homologs (Supplementary Figure S3). Considering the notable high conservation of paratox within prophages, its distribution across various serotypes was evaluated. However, there was no correlation between the prevalence of paratox and bacterial serotype (Figure 5B). This lack of correlation was also observed when examining clonal complexes.

Analysis of the GBS host genomes revealed the presence of another *prx* homolog in the chromosome of every GBS clinical isolate in this study. The host genome-encoded *prx* genes were adjacent to a common set of genes found in every bacterial strain; however, they were not the same genes found adjacent to the prophage-encoded *prx* gene. The region surrounding the *prx* gene on the host genome begins with an integrase and ends with the *prx* gene flanked by two attachment sites (Figure 5C). This region also encodes transposase elements and several bacterial genes, suggesting that this may be a mobile genetic element. An amino acid alignment of these host genome-encoded paratox proteins with the prophage paratox proteins showed some conservation of the amino acids (Supplementary Figure S3). However, the host-encoded paratox protein contains three additional amino acids at the N-terminus than the canonical prophage paratox protein.

### 3.6. GBS Prophages Encode a Gene Upstream of Prx with Holin-like and Transmembrane Domains

In the *S. pyogenes* prophages that encode paratox, the *prx* gene is always located adjacent to a toxin-encoding gene, and prophages lacking a toxin-encoding gene also lack the *prx* gene [66]; therefore, the name para-tox (adjacent to a toxin) is used. To investigate whether the *prx* gene is adjacent to a toxin-encoding gene in the GBS prophages, the region surrounding *prx* was analyzed. Except for cluster B and a few cluster A prophages, all prophages carrying the *prx* gene have an ORF encoding a putative holin-like gene (previously designated as *holtox* [67]) adjacent to and divergently transcribed from the *prx* gene (Figure 4 and Figure 5A). The function of the protein encoded by the *holtox* gene is not known, but bioinformatic analyses reveal the presence of a holin domain (PF16935). The alignment of *prx* genes reveals that two genes encoding a hypothetical protein and a putative *holtox* are typically adjacent to and divergently transcribed from the *prx* genes in clusters A and C (Figure 5A). All cluster D and E prophages lack the *prx* genes. Notably, all but three cluster D prophages encode the *holtox* gene, which suggests that *prx* and *holtox* genes are not likely inherited as a single module (Figure 4).

## 4. Discussion

Prophages play a major role in virulence in many pathogens, including *Streptococcal* pathogens. *S. pyogenes* requires multiple prophage-encoded virulence genes for successful infection [68]. While colonization by *S. pyogenes* always has the potential to result in disease, GBS can colonize the human urogenital tract and behave as a commensal, only becoming a pathogen under certain circumstances. In particular, GBS colonization of the vaginal tract of pregnant women is a major risk factor for transmission to the neonate, resulting in life-threatening disease. However, little is known about the role or the presence of prophages in this opportunistic pathogen. Therefore, this study utilized a collection of 42 GBS human vaginal isolates from pregnant women, which had the potential to cause neonatal disease, to determine the presence and diversity of prophages. The relationship between prophages and previously described GBS virulence genotypes was investigated. Seven previously analyzed GBS clinical strains were added to the investigation as reference strains.

There is a high level of prophage diversity among the GBS strains, with most strains having at least one prophage in their genome and some strains carrying multiple prophages. This is consistent with other studies on GBS prophages [26,27,28,29]. Out of the 49 strains investigated, 41 (~84%) had at least one prophage in their genome, with 6 strains carrying two complete prophage genomes. Serotypes Ia, III, and V, linked to GBS invasive disease [56,57], had a higher proportion of prophages. However, this may be due to the fact that most of the bacterial genomes analyzed in this study belonged to these three serotypes, explaining why over 70% of the prophages identified were found in these serotypes. Additionally, there was no relationship between serotype and the presence/absence of prophages.

Cohabiting prophages can work together to regulate bacterial gene expression, prophage induction, and decrease antibiotic sensitivity [69]. In our dataset, 90% of bacterial strains with multiple prophages carried a cluster A and a cluster D prophage, with one always from cluster A. Carrying multiple prophages from varying clusters increases bacterial genetic diversity and may reinforce bacterial fitness and virulence. This could occur through gene expression complementation or one prophage activating another prophage [70,71]. However, it is unclear whether prophages interact in poly lysogenic GBS, and further research is needed to test these hypotheses.

Integration of prophages into bacterial genomes also has the potential to impact bacterial gene expression. The majority of prophages encoding tyrosine integrases integrate into the 3′ end of tRNAs, and transcriptional orientation of prophage genes adjacent to attL and attR does not appear to affect expression of bacterial genes that flank the prophage genome. GBS prophage integration sites vary among prophage clusters, but prophages within the same cluster tend to integrate into a common site. Prophage integration can disrupt genes, as observed in *Staphylococcus aureus* when lysogenized by phi13 phage, leading to the loss of beta-toxin expression [72]. Conversely, prophage excision can impact bacterial phagosomal escape, as seen in *L. monocytogenes*, where excision of the prophage leads to the expression of the *com* genes and allows for escape from phagosomes [60]. The dataset reported here showed several prophages integrate into the *com* locus, which is in line with other studies on GBS prophages [26,28,55], and further research is needed to understand the relevance of this insertion site on the fitness and virulence of GBS strains.

Prophages can spread virulence genes in pathogenic bacteria and increase bacterial fitness during infection, such as enhancing adhesion to epithelial cells, increasing survival in serum, and improving antibiotic resistance [26,73]. Within our dataset, multiple genes of interest were identified including toxin–antitoxin systems. Four of the six TA systems identified in our study have been previously reported in GBS prophages [26]. Some genes were unique to this study, including a gene with homology to SEFIR/Toll/interleukin-1 receptor domain-containing protein domain and MazG, which is involved in bacterial survival under nutrition stress [74]. Additional studies will be needed to investigate the specific functions of these genes and their role in GBS pathogenesis.

A significant finding was that the *prx* gene, which encodes the paratox protein involved in bacterial competence [32], is present in all 49 clinical isolates of GBS. While the *prx* gene is found in 69% of prophage genomes, it is also found in 100% of the bacterial host chromosomes, meaning that some GBS strains carry multiple copies of paratox. The region containing the *prx* gene on the bacterial host chromosome has a phage integrase located upstream of an attL site at the 5′ end, suggesting that this *prx* gene may be a genomic remnant of a previous phage. The presence of a transposase gene also in this region suggests the *prx* gene may be part of a transposon. Therefore, it is not known whether the *prx* gene on the host chromosome was originally a phage gene or was inherited from a mobile genetic element. However, the high conservation of the *prx* gene, whether prophage encoded or host encoded, suggests having this gene present provides a significant fitness advantage, particularly since all bacterial strains contain the *prx* gene regardless of having a prophage.

Unlike in *S. pyogenes*, the *prx* gene in GBS prophages is not always adjacent to a toxin-encoding gene. In some instances, a putative *holtox* gene is located next to, but divergently transcribed from, *prx*, but it is unclear whether it functions as a toxin. This putative *holtox* is homologous to a holin-like toxin gene that encodes a protein thought to have antibacterial activity against Gram-positive bacteria and complements a lysis defective bacteriophage [67]. Further research into this gene is necessary to provide insight into its function in GBS.

Prophages have been consistently found to contribute to bacterial fitness and virulence. This study investigated the presence and diversity of prophages in 49 human isolates of GBS strains. GBS prophages are very diverse, with most strains carrying at least one prophage and some carrying multiple prophages. Cohabiting prophages tended to have specific combinations of prophage clusters, which could suggest cooperating prophage interactions that may impact bacterial fitness or virulence. Prophages were found to integrate into specific sites, including genes within the *com* locus, which could impact bacterial fitness. Notably, all 49 clinical isolates of GBS had the *prx* gene, which encodes the paradox protein that is involved in bacterial competence in *S. pyogenes*, present on the bacterial host chromosome, and may provide a fitness advantage. Additional research on prophages in GBS is needed, as they may have a significant effect on bacterial fitness and virulence.

## Figures and Tables

**Table 1:** Characteristics of GBS prophages.

Prophage	Cluster	Genome Size (bp)	% GC	No. of Genes	Type of Integrase	Gene Upstream of Insertion Site (Att Site)	Inserts Into	Gene Downstream of Insertion Site (Att Site)	Attachment Site
Javan 5 (2603 V/R)	A	40,574	35.3	78	Tyrosine	integrase	tRNA-Arg	LSU ribosomal protein L19p	ATGTCCCCTGCC
Javan 7 (A909)	A	37,225	37.1	62	Tyrosine	hypothetical protein	N/A	HU family DNA-binding protein	TTATAGTTGGGGCGAATTTGGGGCATAA
prophigbs515	A	40,634	34.9	89	Tyrosine	integrase	tRNA-Arg	LSU ribosomal protein L19p	ATGTCCCCTGCC
prophiDMC2-1	A	39,700	36.8	66	Tyrosine	integrase	tRNA-Arg	LSU ribosomal protein L19p	ATGTCCCCTGCC
prophiDMC5	A	46,132	35.9	80	Tyrosine	integrase	tRNA-Arg	LSU ribosomal protein L19p	ATGTCCCCTGCC
prophiDMC15	A	38,551	36.7	68	Tyrosine	integrase	tRNA-Arg	LSU ribosomal protein L19p	ATGTCCCCTGCC
prophiDMC16	A	43,746	36.4	71	Tyrosine	integrase	tRNA-Arg	LSU ribosomal protein L19p	ATGTCCCCTGCC
prophiDMC21-2	A	43,397	36.6	73	Serine	N-acetylmannosamine kinase	acetyl xylan esterase	Sialic acid utilization regulator, RpiR family	GATTTTGATGACTTC
prophiDMC25	A	38,551	36.7	68	Tyrosine	integrase	tRNA-Arg	LSU ribosomal protein L19p	ATGTCCCCTGCC
prophiDMC33-1	A	43,746	36.4	72	Tyrosine	integrase	tRNA-Arg	LSU ribosomal protein L19p	ATGTCCCCTGCC
prophiDMC34	A	37,294	36.7	62	Serine	ComGF	N/A	ComGB	TAAATTTTTC
prophiDMC43-1	A	43,738	38.6	58	Tyrosine	bacterial ribosome SSU maturation protein RimP	tRNA-Ser-GGA	tRNA (guanine(46)-N(7))-methyltransferase	AATCCCCTCCTCTCCTTT
prophiDMC47	A	45,805	35.8	81	Tyrosine	integrase	tRNA-Arg	LSU ribosomal protein L19p	ATGTCCCCTGCC
prophiDMC48	A	45,885	35.8	80	Tyrosine	integrase	tRNA-Arg	LSU ribosomal protein L19p	ATGTCCCCTGCC
prophiDMC49	A	45,685	35.8	84	Tyrosine	integrase	tRNA-Arg	LSU ribosomal protein L19p	ATGTCCCCTGCC
prophiDMC51	A	45,805	35.8	82	Tyrosine	integrase	tRNA-Arg	LSU ribosomal protein L19p	ATGTCCCCTGCC
prophiDMC61	A	45,686	35.8	80	Tyrosine	integrase	tRNA-Arg	LSU ribosomal protein L19p	ATGTCCCCTGCC
prophiDMC64	A	40,022	37	68	Tyrosine	integrase	tRNA-Arg	LSU ribosomal protein L19p	ATGTCCCCTGCC
prophiDMC66-1	A	45,884	35.8	80	Tyrosine	integrase	tRNA-Arg	LSU ribosomal protein L19p	ATGTCCCCTGCC
prophiDMC67	A	37,262	36.8	61	Serine	ComGF	N/A	ComGB	TAAATTTTTC
prophiDMC68-1	A	45,777	35.8	80	Tyrosine	integrase	tRNA-Arg	LSU ribosomal protein L19p	ATGTCCCCTGCC
prophiDMC6	B	36,849	39.7	57	Serine	ComGD	ComGC	ComGB	TAAATTTTTC
prophiDMC17	B	36,582	39.7	54	Serine	ComGD	ComGC	ComGB	TAAATTTTTC
prophiDMC30	B	36,585	39.7	56	Serine	ComGD	ComGC	ComGB	TAAATTTTTC
prophiDMC36	B	36,581	39.6	54	Serine	ComGD	ComGC	ComGB	TAAATTTTTC
prophiDMC62	B	36,534	39.7	54	Serine	ComGD	ComGC	ComGB	TAAATTTTTC
prophiCNCTC 10/84	C	40,696	36.4	67	Tyrosine	hypothetical protein	N/A	HU family DNA-binding protein	TTATAGTTGGGGCGAATTTGGGGCATAA
prophiDMC4	C	38,991	36.2	55	Tyrosine	hypothetical protein	N/A	DNA binding protein HbSu	TTATGCCCCAAATTCGCCCCAACTATAA
prophiDMC9	C	38,963	36.2	55	Tyrosine	hypothetical protein	N/A	DNA-binding protein HbSu	TTATGCCCCAAATTCGCCCCAACTATAA
prophiDMC69	C	38,991	36.2	65	Tyrosine	hypothetical protein	N/A	DNA binding protein HbSu	TTATAGTTGGGGCGAATTTGGGGCATAA
Javan 8 (A909)	D	45,841	42.2	43	Serine	transcriptional regulator AcrR family	N/A	hypothetical protein	ACTTTTGAAAAGGAGA
prophiCJBIII (CJBIII)	D	48,336	41.8	46	Serine	transcriptional regulator AcrR family	N/A	hypothetical protein	ACTTTTGAAAAGGAGA
prophiDMC1	D	45,705	42.5	45	Serine	transcriptional regulator AcrR family	N/A	hypothetical protein	ACTTTTGAAAAGGAGA
prophiDMC2-2	D	46,693	42.5	49	Serine	transcriptional regulator AcrR family	N/A	hypothetical protein	ACTTTTGAAAAGGAGA
prophiDMC21-1	D	45,421	42.5	45	Serine	transcriptional regulator AcrR family	N/A	hypothetical protein	ACTTTTGAAAAGGAGA
prophiDMC24	D	43,168	43.9	46	Serine	hypothetical protein	N/A	hydrolase (HAD superfamily)	TGGTATAAT
prophiDMC28	D	45,702	42.5	45	Serine	transcriptional regulator AcrR family	N/A	hypothetical protein	ACTTTTGAAAAGGAGA
prophiDMC33-2	D	46,690	42.5	45	Serine	transcriptional regulator AcrR family	N/A	hypothetical protein	ACTTTTGAAAAGGAGA
prophiDMC43-2	D	44,915	42.8	44	Serine	transcriptional regulator AcrR family	N/A	hypothetical protein	ACTTTTGAAAAGGAGA
prophiDMC66-2	D	43,168	43.9	47	Serine	hypothetical protein	N/A	hydrolase (HAD superfamily)	TGGTATAAT
Javan 6 (2603 V/R)	E	34,100	40.2	40	Tyrosine	alkyl hydroperoxide reductase protein F	tRNA-Cys	Na+/H+ antiporter	AATCCGTCTACCGCCT
prophiDMC20	E	36,343	40	53	Tyrosine	alkyl hydroperoxide reductase protein F	tRNA-Cys	Na+/H+ antiporter	AATCCGTCTACCGCCT
prophiDMC27	E	36,093	40	53	Tyrosine	alkyl hydroperoxide reductase protein F	tRNA-Cys	Na+/H+ antiporter	AATCCGTCTACCGCCT

## Data Availability

Contigs of bacterial genomes in this study can be found at the NCBI BioProject under accession number PRJNA888223.

## Supplementary Materials

The following supporting information can be downloaded at: https://www.mdpi.com/article/10.3390/pathogens13080610/s1, Figure S1: Comparative analysis between GBS prophage; Figure S2: (A–E): Genome comparisons of cluster prophages; Figure S3: Paratox is highly conserved across clusters; Figure S4: Genes of co-habiting GBS prophages; Table S1: GBS strains and resident prophages; Table S2: Prophage gene content similarity matrix.
