# Supplementary material for: Comparative Genomic Analysis of Prophages in Human Vaginal Isolates of Streptococcus agalactiae
Source: Pathogens. 2024 Jul 23;13(8):610. doi: 10.3390/pathogens13080610 (PMC11357604; doi:10.3390/pathogens13080610)
Supplement: Supplementary file 1 [file pathogens-13-00610-s001.zip › pathogens-3092555-supplementary.pdf]

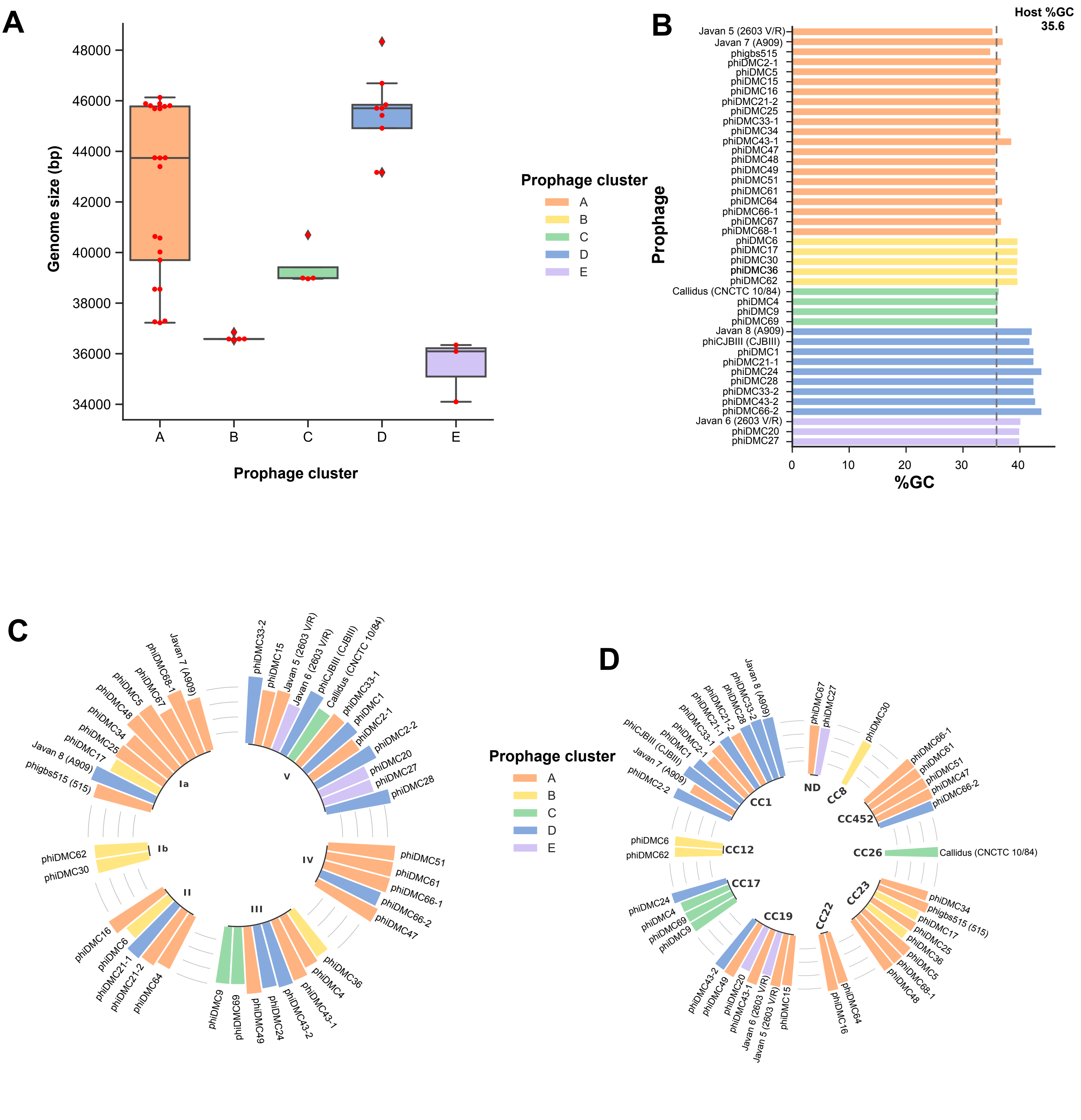

Supplemental Figure 1: Comparative analysis between GBS prophage genomes

A. Genome size distribution of prophages from GBS clinical isolates varies among different clusters. B. The average %GC content of prophages from GBS clinical isolates differs among the different clusters and were mostly higher than the streptococcal host. Dotted line indicates %GC content of the host. C. Prophage cluster and genome size distribution across the different serotypes. D. Organization of prophage genomes by clonal cluster of the host

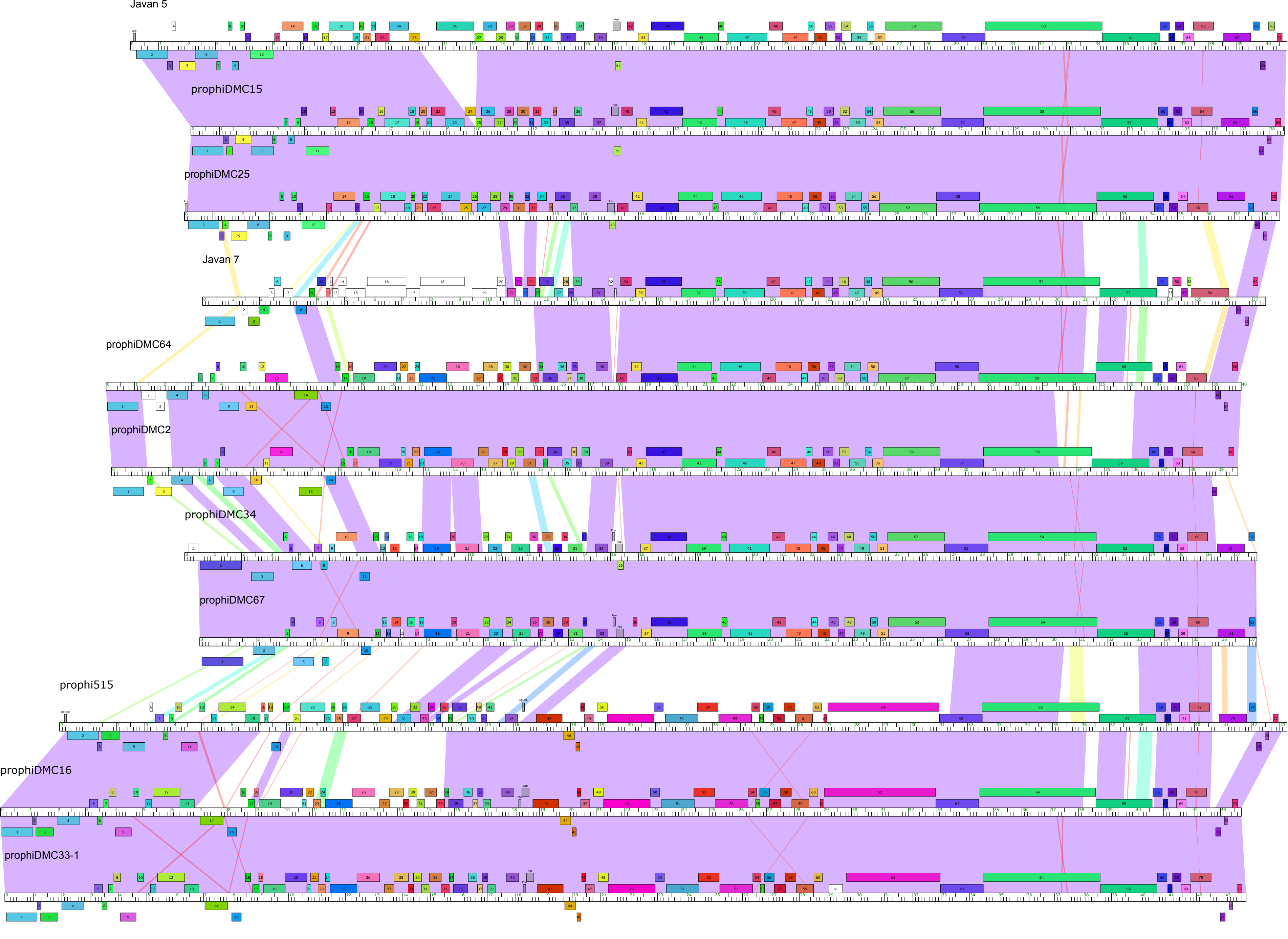
Javan 5
prophiDMC15
prophiDMC25
Javan 7
prophiDMC64
prophiDMC2
prophiDMC34
prophiDMC67
prophi515
prophiDMC16
prophiDMC33-1

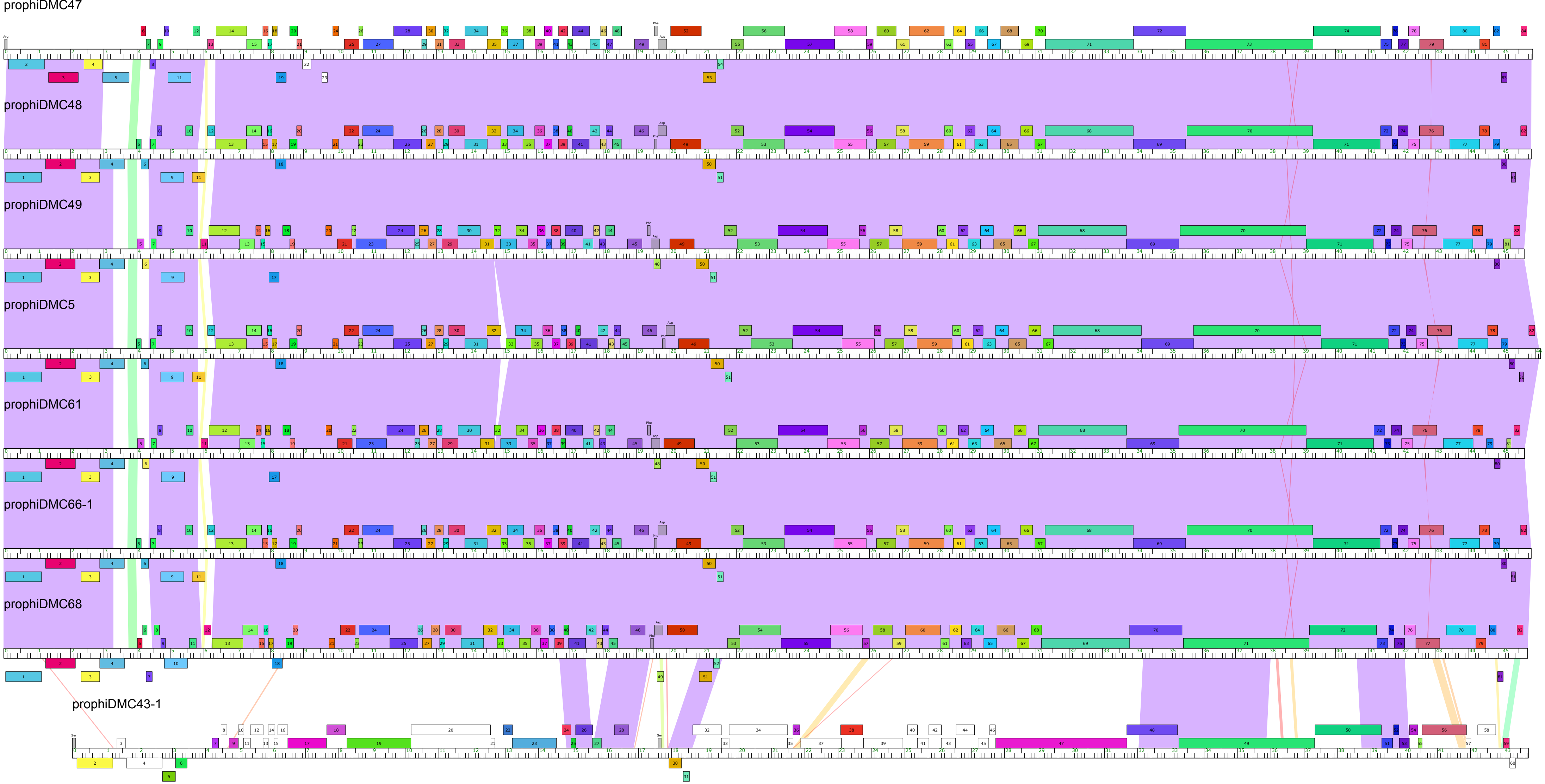

Supplemental Figure 2A: Genome comparisons of Cluster A prophages. Genome similarity in nucleotide sequence is illustrated using a color spectrum. The colors range from violet to red, where violet represents the highest similarity and red the lowest. Genes are represented as colored boxes, positioned above or below each genome, which indicate their direction of transcription. The color of the boxes corresponds to their gene phamilies. The maps were generated using phamerator software and the Streptococcus agalactiae (version 1) database.

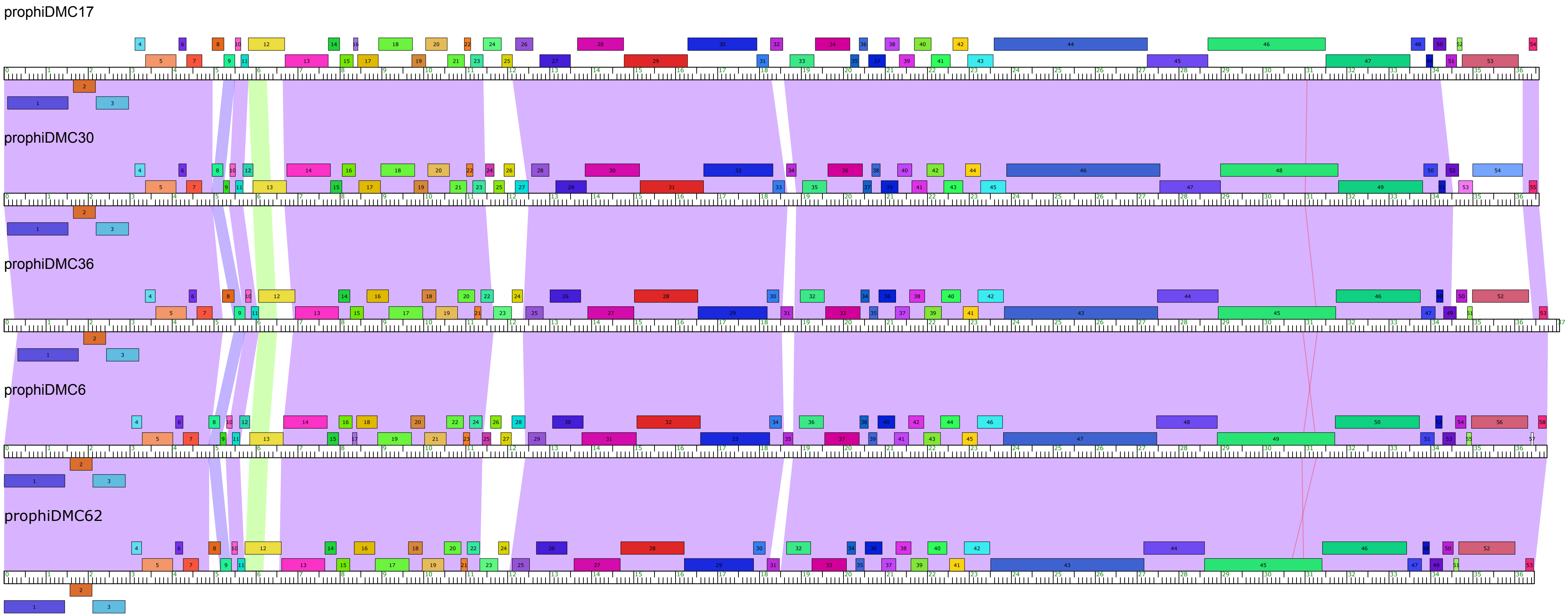

Supplemental Figure 2B: Genome comparisons of Cluster B prophages. Genome similarity in nucleotide sequence is illustrated using a color spectrum. The colors range from violet to red, where violet represents the highest similarity and red the lowest. Genes are represented as colored boxes, positioned above or below each genome, which indicate their direction of transcription. The color of the boxes corresponds to their gene phamilies. The maps were generated using phamerator software and the Streptococcus agalactiae (version 1) database.

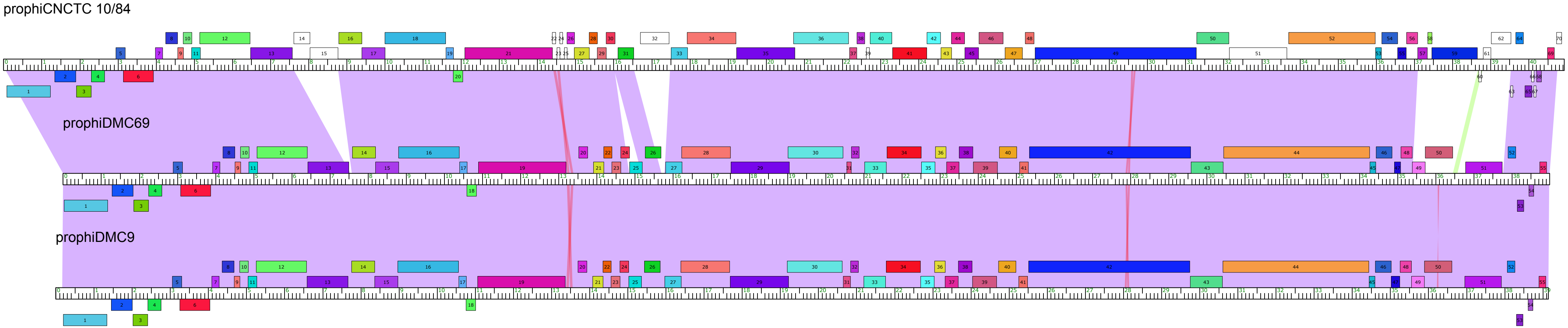

Supplemental Figure 2C: Genome comparisons of Cluster C prophages. Genome similarity in nucleotide sequence is illustrated using a color spectrum. The colors range from violet to red, where violet represents the highest similarity and red the lowest. Genes ar represented as colored boxes, positioned above or below each genome, which indicate their direction of transcription. The color of the boxes corresponds to their gene phamilies. The maps were generated using phamerator software and the Streptococcus agalactiae (version 1) database.

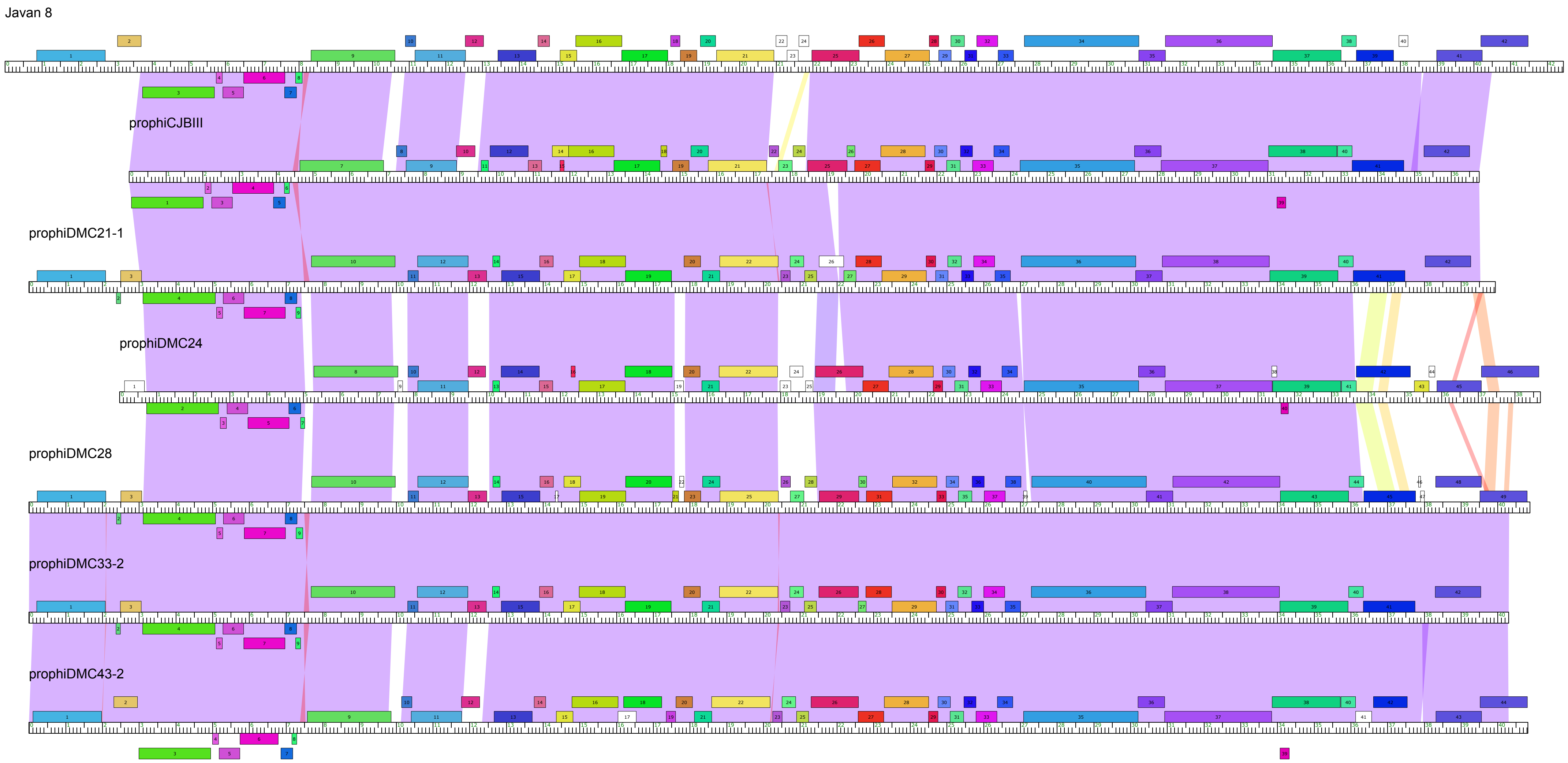

Supplemental Figure 2D: Genome comparisons of Cluster D prophages. Genome similarity in nucleotide sequence is illustrated using a color spectrum. The colors range from violet to red, where violet represents the highest similarity and red the lowest. Genes are represented as colored boxes, positioned above or below each genome, which indicate their direction of transcription. The color of the boxes corresponds to their gene phamilies. The maps were generated using phamerator software and the Streptococcus agalactiae (version 1) database.

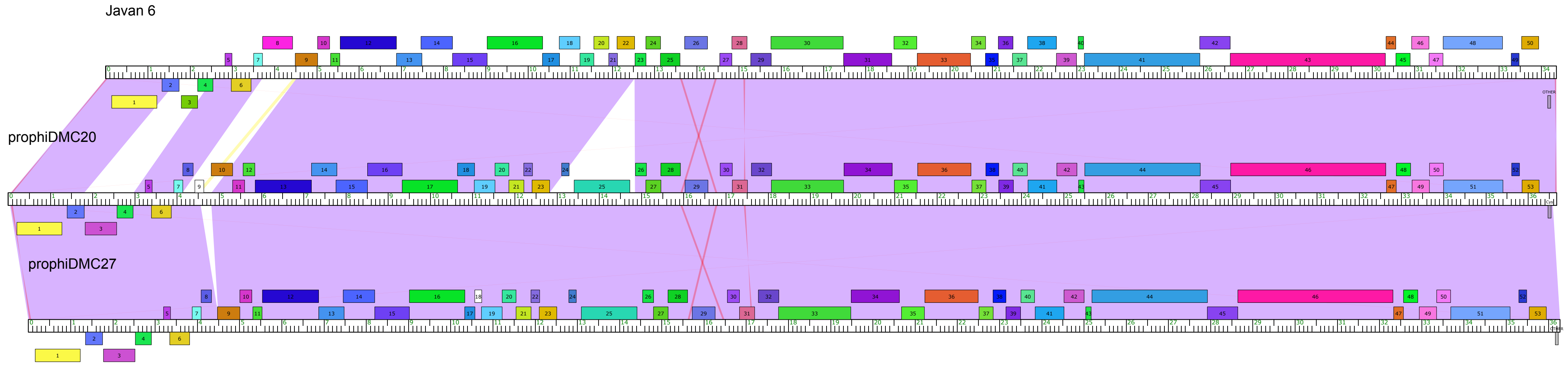

Supplemental Figure 2E: Genome comparisons of Cluster E prophages. Genome similarity in nucleotide sequence is illustrated using a color spectrum. The colors range from violet to red, where violet represents the highest similarity and red the lowest. Genes are represented as colored boxes, positioned above or below each genome, which indicate their direction of transcription. The color of the boxes corresponds to their gene phamilies. The maps were generated using phamerator software and the Streptococcus agalactiae (version 1) database.

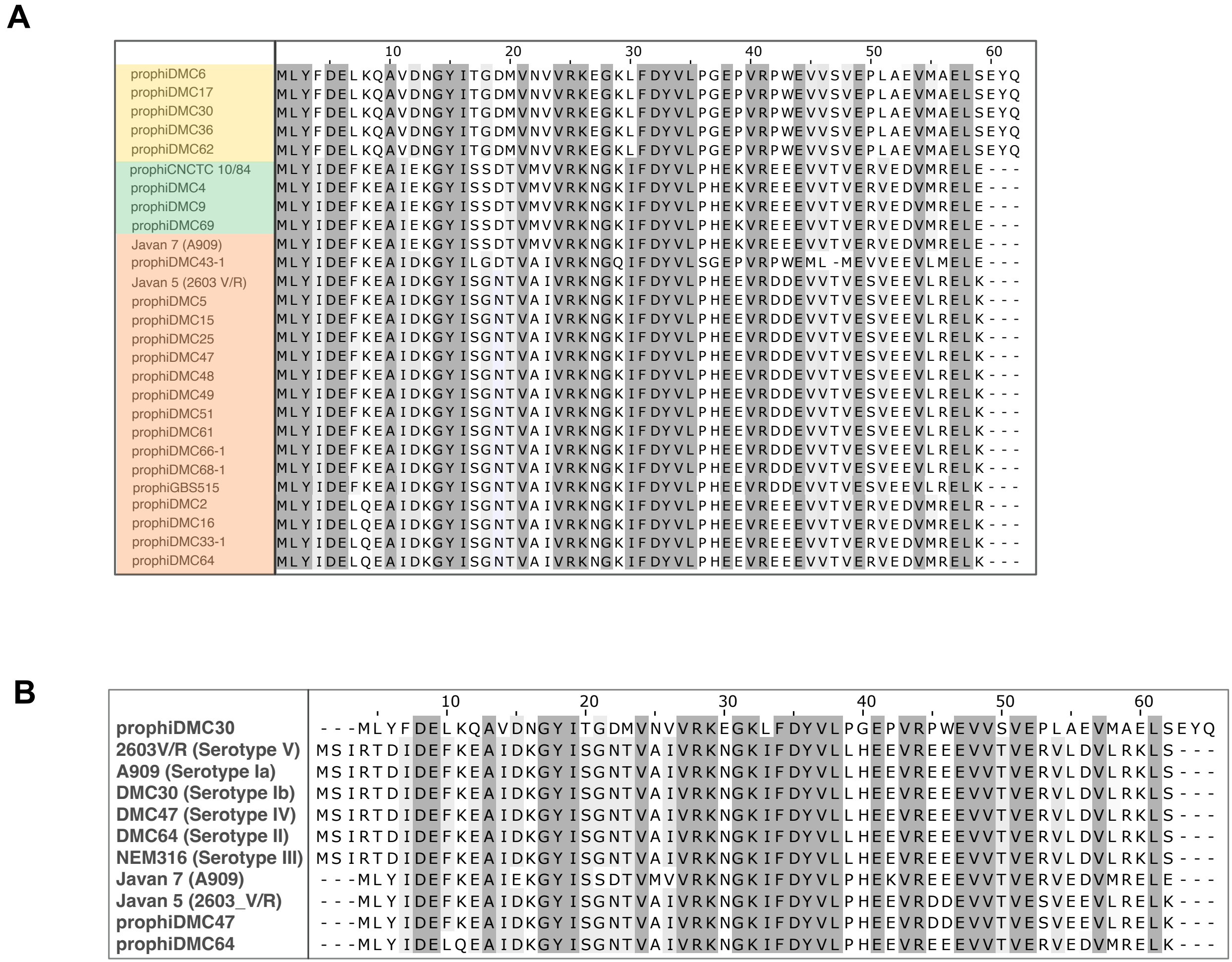

Supplemental Figure 3: Paratox is highly conserved across clusters

A. Clustal alignment of prophage paratox amino acid sequence by cluster. B. Clustal alignment of amino acid sequences of the host paratox and prophage paratox proteins.

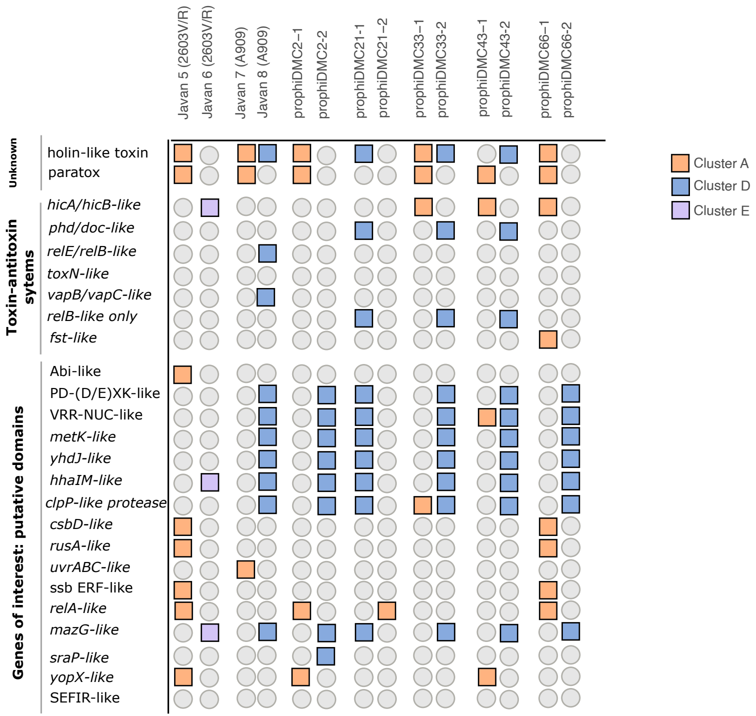

Supplemental Figure 4. Genes of co-habiting GBS prophages.

Solid colored boxes represent the presence of a gene by prophage cluster. Absence of either gene is indicated by a grey circle.

Supplemental Table 1: GBS strains and resident prophages.

Bacterial strain	Serotype	Sequence type (ST)	Clonal complex (CC)	Bacterial accession number/contig number	Prophage	Coordinates
2603 V/R	V	110	19	NC_004116.1	Javan 5	558 773 - 599 346
					Javan 6	1 833 089 - 1 867 188
515	Ia	23	23	NZ_CP051004	prophiGBS515	558 712 - 599 345
A909	Ia	7	1	NC_007432	Javan 7	548 935 - 586 159
					Javan 8	654 882 - 700 722
CJBIII	V	1	1	NZ_CP063198	prophiCJBIII	610 250 - 658 585
CNCTC 10/84	V	26	26	NZ_CP006910	prophiCNCTC10/84	550 755 - 591 450
COHI	III	17	17	NZ_HG939456	none[a]	n/a
NEM316	III	23	23	NC_004368.1	none	n/a
DMC 1	V	1	1	NODE_1_length_599266_cov_280.701108 - NODE_4_length_161306_cov_318.978626	prophiDMC1*	1 - 41877/ 157 477 - 161 306
DMC 2	V	827	1	NODE_3_length_166000_cov_356.650576	prophiDMC2-1	19 476 - 59 175
				NODE_14_length_42782_cov_314.025741 - NODE_1_length_600106_cov_289.469613	prophiDMC2-2*	38 596 - 42 783/ 1- 42 863
DMC 3	Ib	12	12	n/a	none	n/a
DMC 4	III	17	17	NODE_6_length_126496_cov_105.233340	prophiDMC4**	69 292 - 108 282
DMC 5	Ia	23	23	NODE_5_length_122198_cov_172.878489/ NODE_1_length_553758_cov_158.382558	prophiDMC5	107 152 - 122 203/ 1 - 31 081
DMC 6	II	12	12	NODE_1_length_322061_cov_150.271571	prophiDMC6	20 785 - 57 633
DMC 9	III	17	17	NODE_6_length_126496_cov_152.635282	prophiDMC9	
DMC 10	Ib	268	12	n/a	none	n/a
DMC 13	IV	468	452	n/a	none	n/a
DMC 14	Ib	12	12	n/a	none	n/a
DMC 15	V	1233	19	NODE_12_length_68891_cov_159.687424	prophiDMC15	810 - 39 360
DMC 16	II	22	22	NODE_8_length_60059_cov_158.772108	prophiDMC16	10 346 - 54 091
DMC 17	Ia	23	23	NODE_9_length_96718_cov_105.006781	prophiDMC17	19 150 - 55 731

DMC 20	V	1233	19	NODE_6_length_120704_cov_196.757259 /<br>NODE_10_length_76177_cov_227.733373	prophiDMC20	1 - 4553/<br>44 389 - 76 179
DMC 21	II	1	1	NODE_16_length_40263_cov_177.997160/<br>NODE_13_length_48478_cov_193.092097	prophiDMC21-1	36 437 - 40 264/<br>1 - 41 593
				NODE_6_length_106360_cov_244.738518	prophiDMC21-2	28 465 - 71 862
DMC 24	III	17	17	NODE_35_length_49021_cov_122.545384	prophiDMC24	2 385 - 45 552
DMC 25	Ia	23	23	NODE_1_length_482712_cov_137.953509	prophiDMC25	337 011 - 375 561
DMC 27	V			NODE_14_length_62871_cov_36.570429	prophiDMC27	17 805 - 53 897
DMC 28	V	1	1	NODE_1_length_426519_cov_109.332004/<br>NODE_5_length_161318_cov_122.882475	prophiDMC28	1874 - 44 712/<br>1 - 48 538
DMC 29	III	27	19	n/a	none	n/a
DMC 30	Ib	8	8	NODE_8_length_115828_cov_177.017061	prophiDMC30	60 089 - 96 673
DMC 33	V	1	1	NODE_3_length_170046_cov_150.976059	prophiDMC33-1	19 476 - 63 221
				NODE_14_length_42784_cov_134.518391/<br>NODE_1_length_600713_cov_128.183997	prophiDMC33-2	38 958 - 42 785/<br>1 - 42 862
DMC 34	Ia	23	23	NODE_6_length_123204_cov_146.566808	prophiDMC34	66 758 - 104 051
DMC 36	III	23	23	NODE_6_length_96719_cov_160.926205	prophiDMC36	40 988 - 77 569
DMC 38	IV			n/a	none	n/a
DMC 39	V	1	1	n/a	none	n/a
DMC 43	III	19	19	NODE_10_length_107641_cov_208.298556	prophiDMC43-1	
				NODE_14_length_86051_cov_199.828977/<br>NODE_66_length_13680_cov_174.668634	prophiDMC43-2	44 964 - 86 051/<br>1 - 3827
DMC 47	IV	452	452	NODE_1_length_286846_cov_195.021334	prophiDMC47	107 777 - 153 581
DMC 48	Ia	23	23	NODE_3_length_199465_cov_344.276219	prophiDMC48	46 676 - 92 560
DMC 49	III	19	19	NODE_1_length_50795_cov_123.840070	prophiDMC49	105 097 - 150 781
DMC 51	IV	452	452	NODE_2_length_286846_cov_168.591931	prophiDMC51***	107 777 - 153 581
DMC 52	III	27	19	n/a	unextractable	n/a
DMC 56	II	22	22	n/a	unextractable	n/a
DMC 59	III	19	19	n/a	unextractable	n/a
DMC 61	IV	452	452	NODE_1_length_287056_cov_172.238812	prophiDMC61	133 453 - 179 138
DMC 62	Ib	12	12	NODE_11_length_56240_cov_63.336624	prophiDMC62	431 - 36 964
DMC 64	II	22	22	NODE_18_length_71407_cov_21.310901	prophiDMC64	11 829 - 51 850
DMC 66	IV	468	452	NODE_1_length_286917_cov_106.947613	prophiDMC66-1	133 258 - 179 141
				NODE_19_length_66792_cov_160.058636	prophiDMC66-<br>2****	20 947 - 64 154

DMC 67	Ia	1733	ND	NODE_6_length_96865_cov_139.495390	prophiDMC67	40 454 - 77 715
DMC 68	Ia	23	23	NODE_1_length_651246_cov_138.757236	prophiDMC68-1	498 319 - 544 095
DMC 69	III	17	17	NODE_1_length_260544_cov_83.279271	prophiDMC69	69 291 - 108 281
DMC 70	III	19	19	[c]n/a	[b]unextractable	n/a

* prophiDMC1 same as prophiDMC28
** prophiDMC4 same as prophiDMC9
*** prophiDMC51 same as prophiDMC47
**** prophiDMC66-2 same as prophiDMC24

[a]GBS strains that did not have an intact prophage are indicated under Prophage as ‘none’.
[b]GBS strains where an intact prophage could not be bioinformatically extracted are indicated as ‘unextractable’.
[c]n/a indicates not applicable.

Supplemental Table 2: Prophage gene content similarity matrix.

	Javan 8	Callidus	Javan 5	Javan 6	Javan 7	phigbs515	phigbsCJBIII	phigbsDMC15	phigbsDMC16	phigbsDMC17	phigbsDMC2	phigbsDMC20	phigbsDMC21-1	phigbsDMC24	phigbsDMC25	phigbsDMC27	phigbsDMC28	phigbsDMC30	phigbsDMC33-1	phigbsDMC33-2	phigbsDMC34	phigbsDMC36	phigbsDMC43-1	phigbsDMC43-2	phigbsDMC47	phigbsDMC48	phigbsDMC49	phigbsDMC5	phigbsDMC6	phigbsDMC61	phigbsDMC62	phigbsDMC64	phigbsDMC66-1	phigbsDMC67	phigbsDMC68	phigbsDMC69	phigbsDMC9
Javan 8	1.0000	0.0182	0.0183	0.0698	0.0196	0.0354	0.6939	0.0185	0.0367	0.0215	0.0189	0.0674	0.7292	0.6538	0.0185	0.0674	0.6792	0.0213	0.0367	0.7660	0.0202	0.0217	0.0870	0.7872	0.0165	0.0167	0.0167	0.0167	0.0206	0.0167	0.0217	0.0185	0.0167	0.0202	0.0167	0.0104	0.0104
Callidus	0.0182	1.0000	0.0373	0.0256	0.1000	0.0432	0.0090	0.0455	0.0444	0.0333	0.0382	0.0165	0.0180	0.0175	0.0455	0.0165	0.0171	0.0163	0.0444	0.0180	0.0156	0.0336	0.0667	0.0179	0.0559	0.0638	0.0563	0.0638	0.0323	0.0563	0.0336	0.0455	0.0638	0.0156	0.0563	0.6892	0.6892
Javan 5	0.0183	0.0373	1.0000	0.0171	0.3100	0.3091	0.0091	0.9296	0.1765	0.1182	0.4362	0.0252	0.0182	0.0177	0.9296	0.0252	0.0172	0.1376	0.1765	0.0182	0.4176	0.1193	0.0948	0.0180	0.2500	0.2627	0.2627	0.2627	0.1339	0.2627	0.1193	0.4124	0.2627	0.4176	0.2521	0.0783	0.0783
Javan 6	0.0698	0.0256	0.0171	1.0000	0.0182	0.0331	0.0575	0.0172	0.0431	0.0196	0.0357	0.8727	0.0690	0.0667	0.0172	0.8727	0.0645	0.0396	0.0431	0.0690	0.0092	0.0198	0.0485	0.0682	0.0397	0.0400	0.0400	0.0400	0.0189	0.0400	0.0198	0.0351	0.0400	0.0092	0.0400	0.0396	0.0396
Javan 7	0.0196	0.1000	0.3100	0.0182	1.0000	0.1709	0.0097	0.3265	0.1667	0.0943	0.3913	0.0088	0.0194	0.0189	0.3265	0.0088	0.0183	0.0636	0.1667	0.0194	0.2842	0.0952	0.1321	0.0192	0.1626	0.1736	0.1639	0.1736	0.0909	0.1639	0.0952	0.3978	0.1736	0.2979	0.1639	0.1038	0.1038
phigbs515	0.0354	0.0432	0.3091	0.0331	0.1709	1.0000	0.0263	0.3241	0.6222	0.0932	0.2051	0.0323	0.0351	0.0342	0.3241	0.0323	0.0333	0.1017	0.6044	0.0351	0.1739	0.0940	0.1368	0.0348	0.3684	0.3964	0.3717	0.3964	0.0992	0.3717	0.0940	0.2222	0.3964	0.1739	0.3717	0.0744	0.0744
phigbsCJBIII	0.6939	0.0090	0.0091	0.0575	0.0097	0.0263	1.0000	0.0092	0.0273	0.0213	0.0093	0.0556	0.8261	0.7400	0.0092	0.0556	0.8000	0.0211	0.0273	0.8667	0.0200	0.0215	0.0753	0.8085	0.0082	0.0083	0.0083	0.0083	0.0204	0.0083	0.0215	0.0092	0.0083	0.0200	0.0083	0.0000	0.0000
phigbsDMC15	0.0185	0.0455	0.9296	0.0172	0.3265	0.3241	0.0092	1.0000	0.1880	0.1193	0.4565	0.0168	0.0183	0.0179	1.0000	0.0168	0.0174	0.1389	0.1880	0.0183	0.4222	0.1204	0.0957	0.0182	0.2521	0.2759	0.2542	0.2759	0.1351	0.2542	0.1204	0.4316	0.2759	0.4222	0.2542	0.0885	0.0885
phigbsDMC16	0.0367	0.0444	0.1765	0.0431	0.1667	0.6222	0.0273	0.1880	1.0000	0.1062	0.3980	0.0420	0.0364	0.0354	0.1880	0.0420	0.0345	0.1053	0.9722	0.0364	0.1491	0.1071	0.1416	0.0360	0.2773	0.3017	0.2797	0.3017	0.1026	0.2797	0.1071	0.4184	0.3017	0.1491	0.2797	0.0678	0.0678
phigbsDMC17	0.0215	0.0333	0.1182	0.0196	0.0943	0.0932	0.0213	0.1193	0.1062	1.0000	0.1215	0.0190	0.0213	0.0206	0.1193	0.0190	0.0200	0.7869	0.1062	0.0213	0.1515	0.9815	0.1089	0.0211	0.0976	0.0984	0.0984	0.0984	0.8667	0.0984	0.9815	0.1193	0.0984	0.1515	0.0984	0.0283	0.0283
phigbsDMC2	0.0189	0.0382	0.4362	0.0357	0.3913	0.2051	0.0093	0.4565	0.3980	0.1215	1.0000	0.0259	0.0187	0.0182	0.4565	0.0259	0.0177	0.1204	0.3980	0.0187	0.4000	0.1226	0.1071	0.0185	0.2672	0.3036	0.2696	0.3036	0.1171	0.2696	0.1226	0.9143	0.3036	0.4157	0.2696	0.0614	0.0614
phigbsDMC20	0.0674	0.0165	0.0252	0.8727	0.0088	0.0323	0.0556	0.0168	0.0420	0.0190	0.0259	1.0000	0.0667	0.0645	0.0168	0.9630	0.0625	0.0385	0.0420	0.0667	0.0089	0.0192	0.0374	0.0659	0.0388	0.0391	0.0391	0.0391	0.0183	0.0391	0.0192	0.0254	0.0391	0.0089	0.0391	0.0286	0.0286
phigbsDMC21-1	0.7292	0.0180	0.0182	0.0690	0.0194	0.0351	0.8261	0.0183	0.0364	0.0213	0.0187	0.0667	1.0000	0.6415	0.0183	0.0667	0.8367	0.0211	0.0364	0.9535	0.0200	0.0215	0.0860	0.8085	0.0164	0.0165	0.0165	0.0165	0.0204	0.0165	0.0215	0.0183	0.0165	0.0200	0.0165	0.0103	0.0103
phigbsDMC24	0.6538	0.0175	0.0177	0.0667	0.0189	0.0342	0.7400	0.0179	0.0354	0.0206	0.0182	0.0645	0.6415	1.0000	0.0179	0.0645	0.6034	0.0204	0.0354	0.6731	0.0194	0.0208	0.0833	0.6604	0.0160	0.0161	0.0161	0.0161	0.0198	0.0161	0.0208	0.0179	0.0161	0.0194	0.0161	0.0100	0.0100
phigbsDMC25	0.0185	0.0455	0.9296	0.0172	0.3265	0.3241	0.0092	1.0000	0.1880	0.1193	0.4565	0.0168	0.0183	0.0179	1.0000	0.0168	0.0174	0.1389	0.1880	0.0183	0.4222	0.1204	0.0957	0.0182	0.2521	0.2759	0.2542	0.2759	0.1351	0.2542	0.1204	0.4316	0.2759	0.4222	0.2542	0.0885	0.0885
phigbsDMC27	0.0674	0.0165	0.0252	0.8727	0.0088	0.0323	0.0556	0.0168	0.0420	0.0190	0.0259	0.9630	0.0667	0.0645	0.0168	1.0000	0.0625	0.0385	0.0420	0.0667	0.0089	0.0192	0.0374	0.0659	0.0388	0.0391	0.0391	0.0391	0.0183	0.0391	0.0192	0.0254	0.0391	0.0089	0.0391	0.0286	0.0286
phigbsDMC28	0.6792	0.0171	0.0172	0.0645	0.0183	0.0333	0.8000	0.0174	0.0345	0.0200	0.0177	0.0625	0.8367	0.6034	0.0174	0.0625	1.0000	0.0198	0.0345	0.8750	0.0189	0.0202	0.0808	0.7500	0.0156	0.0157	0.0157	0.0157	0.0192	0.0157	0.0202	0.0174	0.0157	0.0189	0.0157	0.0097	0.0097
phigbsDMC30	0.0213	0.0163	0.1376	0.0396	0.0636	0.1017	0.0211	0.1389	0.1053	0.7869	0.1204	0.0385	0.0211	0.0204	0.1389	0.0385	0.0198	1.0000	0.1053	0.0211	0.1500	0.8000	0.0762	0.0208	0.0968	0.0976	0.0976	0.0976	0.8833	0.0976	0.8000	0.1182	0.0976	0.1500	0.0976	0.0377	0.0377
phigbsDMC33-1	0.0367	0.0444	0.1765	0.0431	0.1667	0.6044	0.0273	0.1880	0.9722	0.1062	0.3980	0.0420	0.0364	0.0354	0.1880	0.0420	0.0345	0.1053	1.0000	0.0364	0.1491	0.1071	0.1416	0.0360	0.2773	0.3017	0.2797	0.3017	0.1026	0.2797	0.1071	0.4184	0.3017	0.1491	0.2797	0.0678	0.0678
phigbsDMC33-2	0.7660	0.0180	0.0182	0.0690	0.0194	0.0351	0.8667	0.0183	0.0364	0.0213	0.0187	0.0667	0.9535	0.6731	0.0183	0.0667	0.8750	0.0211	0.0364	1.0000	0.0200	0.0215	0.0860	0.8478	0.0164	0.0165	0.0165	0.0165	0.0204	0.0165	0.0215	0.0183	0.0165	0.0200	0.0165	0.0103	0.0103
phigbsDMC34	0.0202	0.0156	0.4176	0.0092	0.2842	0.1739	0.0200	0.4222	0.1491	0.1515	0.4000	0.0089	0.0200	0.0194	0.4222	0.0089	0.0189	0.1500	0.1491	0.0200	1.0000	0.1531	0.0727	0.0198	0.1750	0.1765	0.1864	0.1765	0.1456	0.1864	0.1531	0.3913	0.1765	0.9355	0.1864	0.0455	0.0455
phigbsDMC36	0.0217	0.0336	0.1193	0.0198	0.0952	0.0940	0.0215	0.1204	0.1071	0.9815	0.1226	0.0192	0.0215	0.0208	0.1204	0.0192	0.0202	0.8000	0.1071	0.0215	0.1531	1.0000	0.1100	0.0213	0.0984	0.0992	0.0992	0.0992	0.8500	0.0992	1.0000	0.1204	0.0992	0.1531	0.0992	0.0286	0.0286
phigbsDMC43-1	0.0870	0.0667	0.0948	0.0485	0.1321	0.1368	0.0753	0.0957	0.1416	0.1089	0.1071	0.0374	0.0860	0.0833	0.0957	0.0374	0.0808	0.0762	0.1416	0.0860	0.0727	0.1100	1.0000	0.0851	0.1301	0.1311	0.1311	0.1311	0.1048	0.1311	0.1100	0.1053	0.1311	0.0727	0.1311	0.0660	0.0660
phigbsDMC43-2	0.7872	0.0179	0.0180	0.0682	0.0192	0.0348	0.8085	0.0182	0.0360	0.0211	0.0185	0.0659	0.8085	0.6604	0.0182	0.0659	0.7500	0.0208	0.0360	0.8478	0.0198	0.0213	0.0851	1.0000	0.0163	0.0164	0.0164	0.0164	0.0202	0.0164	0.0213	0.0182	0.0164	0.0198	0.0164	0.0102	0.0102
phigbsDMC47	0.0165	0.0559	0.2500	0.0397	0.1626	0.3684	0.0082	0.2521	0.2773	0.0976	0.2672	0.0388	0.0164	0.0160	0.2521	0.0388	0.0156	0.0968	0.2773	0.0164	0.1750	0.0984	0.1301	0.0163	1.0000	0.8721	0.8721	0.8721	0.0945	0.8721	0.0984	0.2627	0.8721	0.1750	0.9398	0.0794	0.0794
phigbsDMC48	0.0167	0.0638	0.2627	0.0400	0.1736	0.3964	0.0083	0.2759	0.3017	0.0984	0.3036	0.0391	0.0165	0.0161	0.2759	0.0391	0.0157	0.0976	0.3017	0.0165	0.1765	0.0992	0.1311	0.0164	0.8721	1.0000	0.8824	1.0000	0.0952	0.8824	0.0992	0.3097	1.0000	0.1765	0.8824	0.0887	0.0887
phigbsDMC49	0.0167	0.0563	0.2627	0.0400	0.1639	0.3717	0.0083	0.2542	0.2797	0.0984	0.2696	0.0391	0.0165	0.0161	0.2542	0.0391	0.0157	0.0976	0.2797	0.0165	0.1864	0.0992	0.1311	0.0164	0.8721	0.8824	1.0000	0.8824	0.0952	1.0000	0.0992	0.2650	0.8824	0.1765	0.9277	0.0800	0.0800
phigbsDMC5	0.0167	0.0638	0.2627	0.0400	0.1736	0.3964	0.0083	0.2759	0.3017	0.0984	0.3036	0.0391	0.0165	0.0161	0.2759	0.0391	0.0157	0.0976	0.3017	0.0165	0.1765	0.0992	0.1311	0.0164	0.8721	1.0000	0.8824	1.0000	0.0952	0.8824	0.0992	0.3097	1.0000	0.1765	0.8824	0.0887	0.0887
phigbsDMC6	0.0206	0.0323	0.1339	0.0189	0.0909	0.0992	0.0204	0.1351	0.1026	0.8667	0.1171	0.0183	0.0204	0.0198	0.1351	0.0183	0.0192	0.8833	0.1026	0.0204	0.1456	0.8500	0.1048	0.0202	0.0945	0.0952	0.0952	0.0952	1.0000	0.0952	0.8500	0.1150	0.0952	0.1456	0.0952	0.0367	0.0367
phigbsDMC61	0.0167	0.0563	0.2627	0.0400	0.1639	0.3717	0.0083	0.2542	0.2797	0.0984	0.2696	0.0391	0.0165	0.0161	0.2542	0.0391	0.0157	0.0976	0.2797	0.0165	0.1864	0.0992	0.1311	0.0164	0.8721	0.8824	1.0000	0.8824	0.0952	1.0000	0.0992	0.2650	0.8824	0.1765	0.9277	0.0800	0.0800
phigbsDMC62	0.0217	0.0336	0.1193	0.0198	0.0952	0.0940	0.0215	0.1204	0.1071	0.9815	0.1226	0.0192	0.0215	0.0208	0.1204	0.0192	0.0202	0.8000	0.1071	0.0215	0.1531	1.0000	0.1100	0.0213	0.0984	0.0992	0.0992	0.0992	0.8500	0.0992	1.0000	0.1204	0.0992	0.1531	0.0992	0.0286	0.0286
phigbsDMC64	0.0185	0.0455	0.4124	0.0351	0.3978	0.2222	0.0092	0.4316	0.4184	0.1193	0.9143	0.0254	0.0183	0.0179	0.4316	0.0254	0.0174	0.1182	0.4184	0.0183	0.3913	0.1204	0.1053	0.0182	0.2627	0.3097	0.2650	0.3097	0.1150	0.2650	0.1204	1.0000	0.3097	0.4066	0.2650	0.0696	0.0696
phigbsDMC66-1	0.0167	0.0638	0.2627	0.0400	0.1736	0.3964	0.0083	0.2759	0.3017	0.0984	0.3036	0.0391	0.0165	0.0161	0.2759	0.0391	0.0157	0.0976	0.3017	0.0165	0.1765	0.0992	0.1311	0.0164	0.8721	1.0000	0.8824	1.0000	0.0952	0.8824	0.0992	0.3097	1.0000	0.1765	0.8824	0.0887	0.0887
phigbsDMC67	0.0202	0.0156	0.4176	0.0092	0.2979	0.1739	0.0200	0.4222	0.1491	0.1515	0.4157	0.0089	0.0200	0.0194	0.4222	0.0089	0.0189	0.1500	0.1491	0.0200	0.9355	0.1531	0.0727	0.0198	0.1750	0.1765	0.1765	0.1765	0.1456	0.1765	0.1531	0.4066	0.1765	1.0000	0.1765	0.0455	0.0455
phigbsDMC68	0.0167	0.0563	0.2521	0.0400	0.1639	0.3717	0.0083	0.2542	0.2797	0.0984	0.2696	0.0391	0.0165	0.0161	0.2542	0.0391	0.0157	0.0976	0.2797	0.0165	0.1864	0.0992	0.1311	0.0164	0.9398	0.8824	0.9277	0.8824	0.0952	0.9277	0.0992	0.2650	0.8824	0.1765	1.0000	0.0800	0.0800
phigbsDMC69	0.0104	0.6892	0.0783	0.0396	0.1038	0.0744	0.0000	0.0885	0.0678	0.0283	0.0614	0.0286	0.0103	0.0100	0.0885	0.0286	0.0097	0.0377	0.0678	0.0103	0.0455	0.0286	0.0660	0.0102	0.0794	0.0887	0.0800	0.0887	0.0367	0.0800	0.0286	0.0696	0.0887	0.0455	0.0800	1.0000	1.0000
phigbsDMC9	0.0104	0.6892	0.0783	0.0396	0.1038	0.0744	0.0000	0.0885	0.0678	0.0283	0.0614	0.0286	0.0103	0.0100	0.0885	0.0286	0.0097	0.0377	0.0678	0.0103	0.0455	0.0286	0.0660	0.0102	0.0794	0.0887	0.0800	0.0887	0.0367	0.0800	0.0286	0.0696	0.0887	0.0455	0.0800	1.0000	1.0000
